# Hairy cell leukaemia with unusual *BRAF* mutations

**DOI:** 10.1111/jcmm.17890

**Published:** 2023-08-02

**Authors:** Elsa Maitre, Margaret Macro, Xavier Troussard

**Affiliations:** ^1^ Laboratoire d'Hématologie CHU Caen Normandie Caen France; ^2^ Institut bas Normand d'Hématologie CHU Caen Normandie Caen France

**Keywords:** *BRAF* F595L, *BRAF* V600E, hairy cell leukaemia

## Abstract

Hairy cell leukaemia (HCL) diagnosis is based on the morphologic detection of circulating abnormal hairy cells in the peripheral blood and/or bone marrow, an HCL immunological score of 3 or 4 based on the expression of the CD11c, CD25, CD103 and CD123 and also the presence of a *BRAF V600E* activating mutation in the B‐raf proto‐oncogene (BRAF gene) (7q34). When using new generation sequencing of 21 targeted genes in 124 HCL patients, we identified a cohort of 6/124 (2%) patients with unusual *BRAF* mutations: two patients presented non‐V600 mutations (BRAF F595L, BRAF W604L respectively) and four other patients silent BRAF mutations. When using droplet digital PCR (ddPCR) three of the four patients with concomitant *BRAF V600E* and silent mutation were negative. The respective role of these mutations in the occurrence of HCL or its progression remains to be clarified, but *BRAF* sequencing is necessary in case of negative *BRAF V600E* by ddPCR.

## 1 INTRODUCTION

Initially described in 1958, hairy cell leukaemia (HCL) is a well‐defined entity. The diagnosis is based on morphological evidence of circulating abnormal hairy cells, an HCL immunological score of 3 or 4 based on the expression of the CD11c, CD25, CD103 and CD123 and also the presence of *BRAF*V600E activating mutation in the B‐raf proto‐oncogene.^1^ Bone marrow biopsy and immunohistochemistry can be helpful for (differential) diagnosis, especially the positivity for Annexin A1. The different *BRAF* mutations that have been identified in patients with solid tumours (malignant melanomas, colorectal or non‐small‐cell lung cancers) are mainly located either in exon 11 or 15 of *BRAF*. Three subgroups of *BRAF* mutations were reported: *BRAF* V600 mutations, which include BRAF V600E/K and V600 other than V600E/K, and non‐V600 mutations.^2^, ^3^

## 2 METHODS

When using new generation sequencing of 21 targeted genes (previously described in Maitre et al.^4^ and Table S1) in 124 HCL patients, we identified a cohort of 6/124 (2%) patients with non‐usual *BRAF* mutations. Non‐V600 mutations (*BRAF* F595L and *BRAF* W604L) were identified in two patients and the other four patients had silent *BRAF* mutations (Table 1). The study was conducted in accordance with and approved by the Institutional Review Board of CCTIRS and CNIL (protocol code 16.405 and date of approval 15 June 2016 and 916367 17 July 2017). *KRAS* and *NRAS* (exons 2 and 3) were subsequently sequenced in patient relapse sample UPN50. Copy number analysis of BRAF zygosity was performed as previously described in Boeva et al.,^5^ by using coverage data normalized to DNA samples from eight healthy patients. Droplet digital PCR (ddPCR) was performed on QX200 system (Bio‐rad) with ddPCR BRAF V600 screening kit (#12001037) in accordance with the manufacturer's specifications. Oral informed consent was made by physicians, and non‐opposition consents was obtained. The study was conducted in accordance with and approved by the Institutional Review Board of CCTIRS and CNIL (protocol code 16.405 and date of approval 15 June 2016 and 916367 17 July 2017).

**TABLE 1:** Alternative BRAF mutations in the cohort of 124 HCL patients.

UPN	Sex	Age^a^	Samples	IGVH status	% Homology	Repertory	ddPCR BRAFV600E (FA)	*NGS : BRAF* (VAF)	*KLF2* (VAF)
UPN‐50	M	57	PB	Mutated	95.7	3–7	Negative	c.1783T>C (p.F595L) (33%)	Splicing mutation c.76‐1G>A (10%) + c.882C>T (p.R294R) (9%)^b^
UPN‐142	M	39	PB	Mutated	97.08	3–49	10.99%	c.T1799A‐(p.V600E) (12%) + c.1811G>T (p.W604L) (12%)	c.884C>A (p.T295K) (1%)^b^
UPN‐109	F	45	BM	Mutated	96.8	4–61	Negative	c.T1799A‐(p.V600E) (4%) + c.1794T>A (p.A598=) (4%)	
UPN‐113	F	52	PB	Mutated	92.1	1–18	Negative	c.T1799A‐(p.V600E) (1%) + c.1788T>C (p.G596=) (1%)	
UPN‐131	M	40	PB	Mutated	92.9	4–34	4.02%	c.T1799A‐(p.V600E) (6%) + c.1794T>C (p.A598=) (6%)	
UPN‐134	M	33	PB	Mutated	93.4	1–69	Negative	c.T1799A‐(p.V600E) (32%) + c.1794T>A (p.A598=) (32%)	c.824G>A (p.S275N) (2%)^b^

Abbreviations: BM, bone marrow; ddPCR, droplet digital PCR; PB, peripheral blood; VAF, variant allele frequency.

^a^ Age at diagnosis.

^b^ Synonymous and/or intronic *KLF2* mutations were associated.

## 3 RESULTS AND DISCUSSION

**FIGURE 1:**
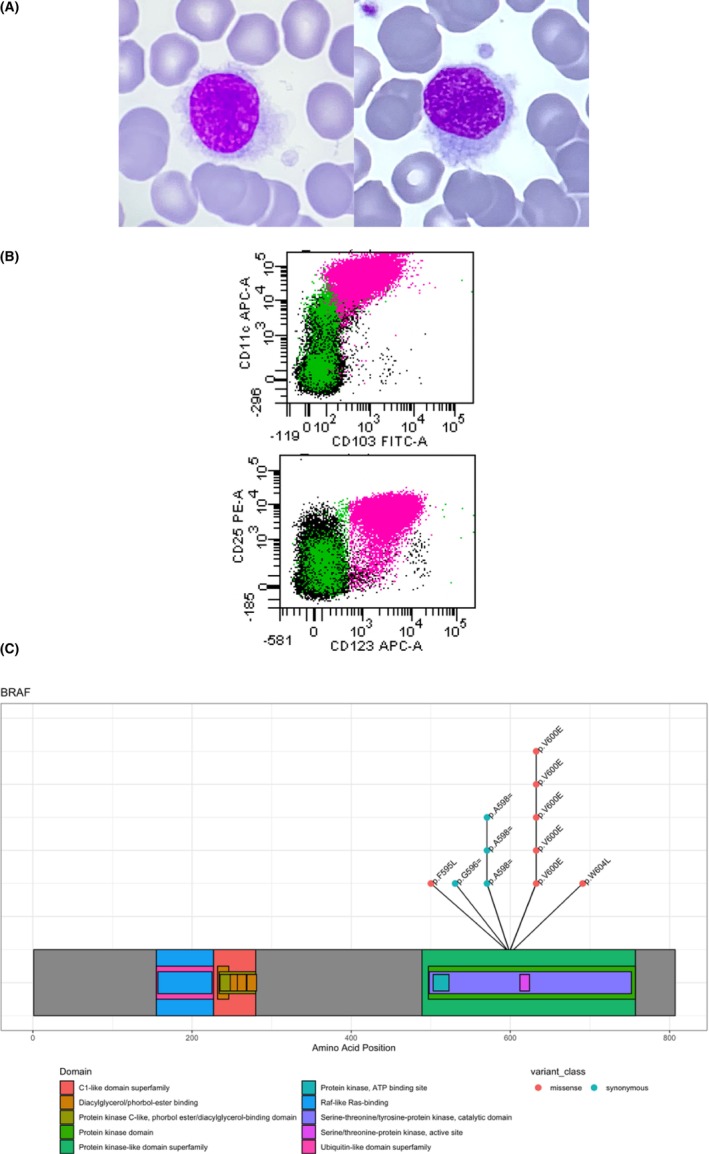
(A) Hairy cells morphology of UPN‐50, May Grunewald Giemsa stain, ×1000; infiltration by 21% of medium size lymphocytes, with regular, round, nucleus, mature chromatin, inconspicuous nucleolus, cytoplasm with long and circumferential villi. (B) Flow cytometry immunophenotype at diagnosis of UPN‐50, pink: Hairy cells, Black: T and NK lymphocytes, Green: residual normal B cells. (C) Lolliplot representation of the BRAF mutations of the patients.

We report for the first time a patient with HCL associated with a *BRAF* F595L mutation. The patient (UPN‐50), a 57‐year‐old man, was hospitalized in September 2011 and presented moderate splenomegaly without peripheral lymph node involvement. The blood cell count showed thrombocytopenia (platelets count: 87 × 10^9^/L), monocytopenia (<0.01 × 10^9^/L), moderate lymphocytosis (6.20 × 10^9^/L) without anaemia (13.4 g/dL) and neutropenia (2.79 × 10^9^/L). The peripheral blood smear analysis detected 33% of typical hairy cells (2.97 × 10^9^/L) (Figure 1A) and the peripheral blood flow cytometry analysis, performed after isolating mononuclear cells by density gradient centrifugation (e.g. peripheral blood mononuclear cells PBMC), showed a clonal B‐cell proliferation (evaluated at 42% of the total number of leucocytes), CD20^bright^, CD79b^bright^ with a kappa light chain restriction. CD5, CD10 and CD38 were negative. The four specific markers of HCL (CD11c, CD25, CD103 and CD123) were brightly expressed (Figure 1B). The bone marrow biopsy was not performed and Annexin A1 staining was not tested. However, the bone marrow infiltration by typical hairy cells and the peripheral blood and bone marrow immunophenotypic analysis were sufficient to establish HCL diagnosis. The activating missense mutation *BRAF* V600E was not detected; however, *BRAF* F595L (c.1783T>C) mutation (Figure 1C, Figure S1) was identified with a variant allele frequency (VAF) of 33%. Copy number analysis of *BRAF* zygosity showed no significant variation suggesting no loss of heterozygosity (Figure S2). Two mutations of Krüppel‐like Factor‐2 (*KLF2*; 19p13) were also associated (Table 1). The immunoglobulin heavy‐chain variable region genes (*IGHV*) were mutated (95.7%) with *VH3‐7* rearrangements. The patient was treated with Cladribine (CDA) in monotherapy but relapsed 8.5 years later in June 2020 with 5.7% of typical hairy cells in the peripheral blood. The *BRAF* F595L (VAF = 4.2%) and *KLF2* mutations were still detected. After further sequencing, no *RAS* (*KRAS*, *NRAS*) mutation was identified. Due to the unusual nature of the mutation and given the doubt on the functional consequences on the degree of the kinase activity, the patient was treated with CDA in monotherapy. A complete response (CR) was achieved and the patient was in CR and asymptomatic in June 2023.

The second patient (UPN‐142) presented on the same allele with atypical *BRAF* V600E associated with *BRAF* W604L (Figure S3). *BRAF* W604L mutation was rarely reported in solid neoplasms (lung adenocarcinoma, malignant melanoma or colorectal carcinoma) but never in healthy individuals (UCSC, gnomAD and ExAc database) and in HCL. The patient, 39‐year‐old, consulted in haematology in July 2021 because of pancytopenia revealed by gastrointestinal infection and fever. The patient did not present hepatosplenomegaly or peripheral lymph node involvement. The blood cell counts showed thrombocytopenia (platelets count: 100 × 10^9^/L), monocytopenia (0.05 × 10^9^/L), anaemia (11.3 g/dL), neutropenia (1.18 × 10^9^/L) and normal lymphocyte count (3.36 × 10^9^/L). The peripheral flow cytometry analysis performed on PBMC showed a clonal B‐cell proliferation (evaluated at 22% of white blood cells), CD20^bright^ and CD79b^bright^ with a kappa light chain restriction. CD5, CD10 and CD38 were negative. HCL‐specific markers (CD11c, CD25, CD103 and CD123) were brightly expressed. A first‐line treatment combining CDA and rituximab was performed according to Chihara et al.^6^: A CR was achieved and persisted at the last follow‐up for 12 months.

Silent *BRAF* mutations were identified in four patients and were located in positions 598 and 596. Interestingly, all synonymous *BRAF* mutations were carried on the same allele with a similar VAF (Figure S4). When using specific *BRAF V600E* ddPCR assay, three of the four patients with BRAF V600E associated with silent mutation were negative (Table 1). The three patients had c.1788T>C (p.596=) and c.1794T>A (p.A598=). As the two mutations are present on the same allele, the hybridization was probably altered, explaining the false‐negative results. Unlike the c.1794T>A (p.A598=), the. c.1794T>C (p.598=) mutation does not seem to impair the detection assay. If specific *BRAF V600E* ddPCR is negative, extensive sequencing of exons 11 and 15 of *BRAF* should be performed for detecting alternative *BRAF* mutations.

*BRAF* V600E mutation is the molecular hallmark in HCL. The absence of *BRAF* V600E was reported in up to 5% of HCL cases.^7^ In those cases, the possibility of non‐V600E *BRAF* mutations should be excluded. The *BRAF* mutations were categorized into three classes based on kinase activity, RAS dependency and dimerization profile of the different mutants. The class I mutations (like *BRAF* V600E) are *BRAF* mutations with a high kinase activity, a mutation of acid V600, an RAS independence and require BRAF monomers. The class II (L597P/Q/R/S, K6001E, …) are also RAS independent and present a high or intermediate kinase activity but require BRAF dimers. Finally, class III mutations (A598V, …) have a kinase‐impaired activity but maintain increased signalling of the MAPK pathway due to enhanced RAS binding and CRAF activation.^8^ In a study analysing 22 mutants, seven were classified in the class I, 11 in the class II and four in the class III.^9^ In a study including 1027 solid tumours, 135 patients (7.6%) presented a *BRAF* mutation: (101/135) of all *BRAF* mutants were kinase activated, 15% kinase impaired (20/135) and 10% kinase unknown (14/135). As expected, the most common mutation was *BRAF* V600E identified in 64% (86/135) but no case of *BRAF* F595L was reported.^10^ The most common kinase‐impaired class III mutation involved codon 594 and was observed in 11% of cases (15/135), specifically D594E/G/H or D594N mutations. Contrary to *BRAF* V600E mutation, these mutations can be associated with activating *RAS* mutations. It is crucial to detect all these mutations by using appropriate methods^11^ and to adapt the treatment, some *BRAF* mutants might not be sensitive to BRAF inhibitors and others sensitive to MEK inhibitors.^3^, ^8^

Only three HCL patients with non‐V600 mutations were previously reported: two patients with mutations in exon 11 (F468C, D449E) and one patient presenting a *BRAF* V600E mutation associated with *BRAF* S602T.^12^ The *BRAF* F595L mutation, disrupting the D^594^F^595^G^596^ motif of the BRAF kinase domain essential for catalysis, was reported in a patient with histiocytic sarcoma. The authors demonstrated the mutation was associated with an intermediate kinase activity and the cooperation with *HRAS* Q61 allowed the promotion of oncogenic signals.^13^ The HCL patient with a *BRAF* F595L mutation located in exon 15 responded to CDA in monotherapy. Note also that the patient does not present any associated poor prognostic factors. The *IGHV* profile was mutated (95.7%) and *VH3‐7* rearrangements were used. In HCL, an unmutated (UM) *IGHV* profile and the VH4‐34 usage is usually associated with resistance to CDA and shorter progression‐free survival and overall survival.^14^, ^15^ The second HCL patient we reported presented a *BRAF* V600E mutation associated with W604L. The patient was treated with CDA plus rituximab and a CR was achieved.

The role of synonymous mutations remains to be studied in HCL. In a study of 659,194 synonymous (silent) mutations from mainly solid tumour samples, including melanoma, the silent mutations were the second most frequent type of point mutations (23.4%) after missense mutations (64.1%).^16^ Synonymous mutations do not change the amino acid sequence and most of them are expected to be functionally neutral. The clinical impact of synonymous mutations remains to be specified but they can change protein levels or protein conformation by altering splicing regulatory sites, mRNA stability, miRNA binding sites or translation efficiency.^17^ Either in or out of the coding regions, they can affect gene expression and may be contribute to tumorigenesis and cancer‐cell fitness.^18^ Note that in our cohort, synonymous mutations were associated in all cases with a *BRAF* V600E mutation. When using ddPCR, alternative *BRAF* mutations could be misdetected due to impaired hybridization and the specificity of the test, which is not able to screen for non‐V600 additional *BRAF* mutations. The respective role of these mutations in the occurrence of HCL or its progression remains to be clarified.

## AUTHOR CONTRIBUTIONS

**Elsa Maitre:** Conceptualization (equal); data curation (equal); formal analysis (lead); investigation (lead); methodology (equal); project administration (equal); validation (equal); visualization (lead); writing – original draft (supporting); writing – review and editing (equal). **Margaret Macro:** Investigation (equal); resources (equal); writing – review and editing (equal). **Xavier Troussard:** Conceptualization (equal); data curation (equal); formal analysis (supporting); investigation (supporting); methodology (equal); project administration (lead); resources (equal); supervision (lead); visualization (supporting); writing – original draft (lead); writing – review and editing (equal).

## CONFLICT OF INTEREST STATEMENT

The authors declare no conflicts of interest.

## Supporting information


Figure S1

Figure S2

Figure S3

Figure S4

Table S1

## Data Availability

The data that support the findings of this study are available from the corresponding author upon reasonable request.
