# Supplementary figures and images for: Hairy cell leukaemia with unusual BRAF mutations
Source: J Cell Mol Med. 2023 Aug 2;27(17):2626–30. doi: 10.1111/jcmm.17890 (PMC10468650; doi:10.1111/jcmm.17890)

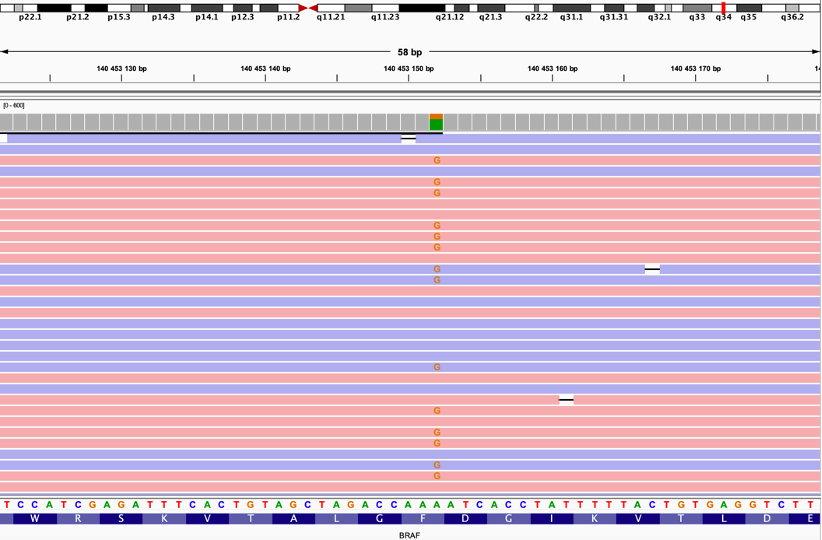

Supplement: Supplementary file 1 — Figure S1 [file JCMM-27-2626-s001.tiff]

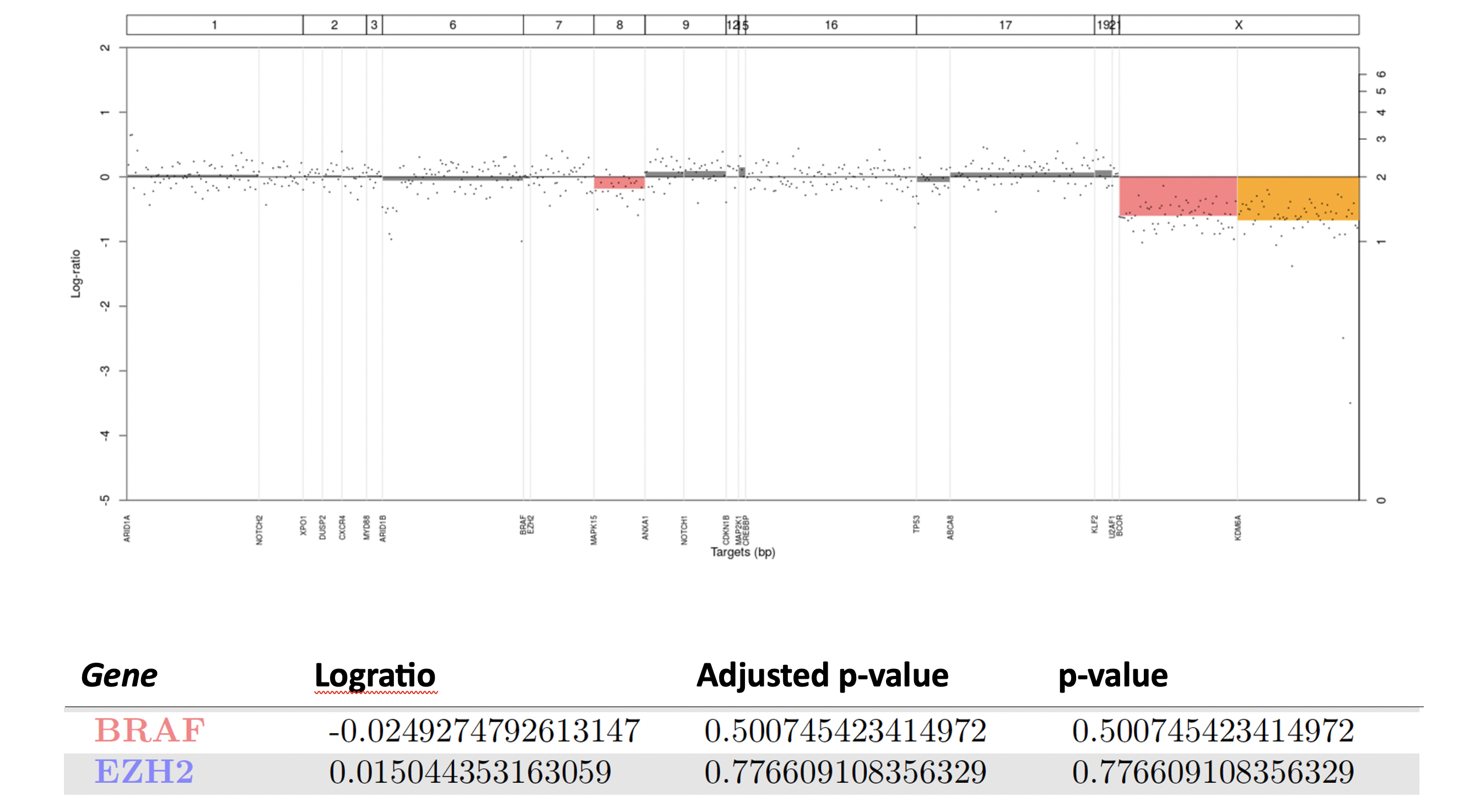

Supplement: Supplementary file 2 — Figure S2 [file JCMM-27-2626-s005.png]

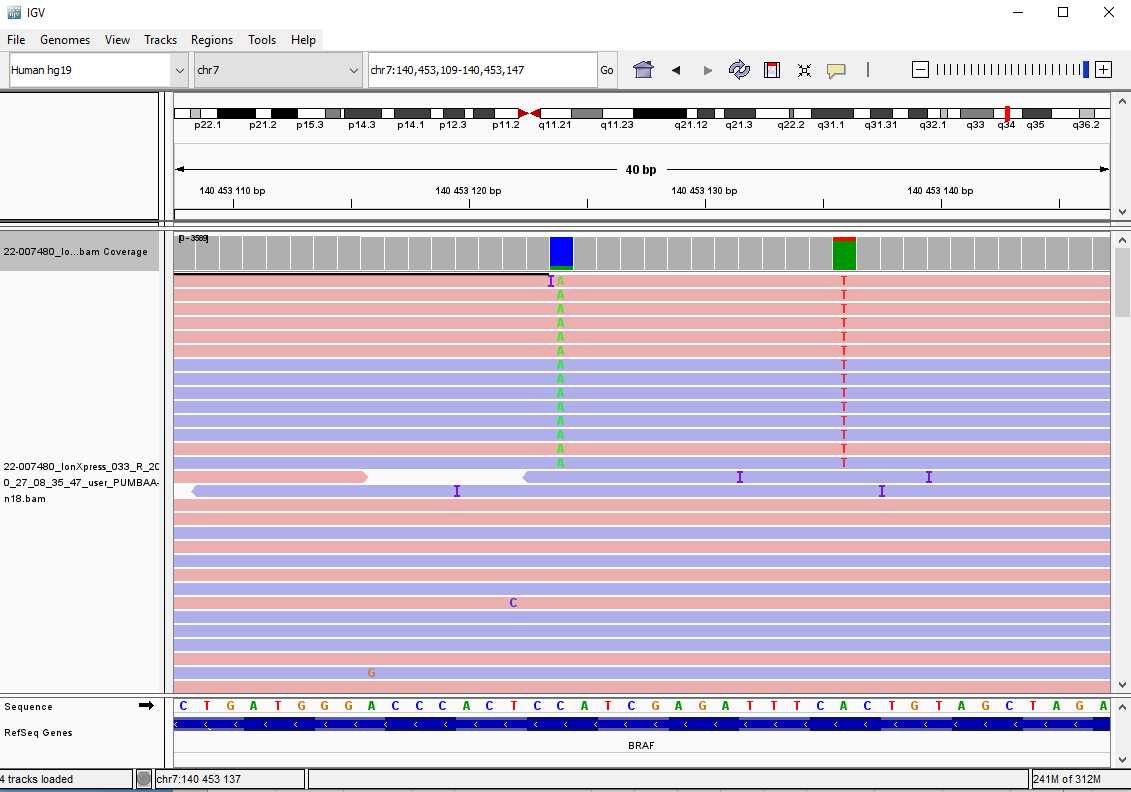

Supplement: Supplementary file 3 — Figure S3 [file JCMM-27-2626-s002.png]

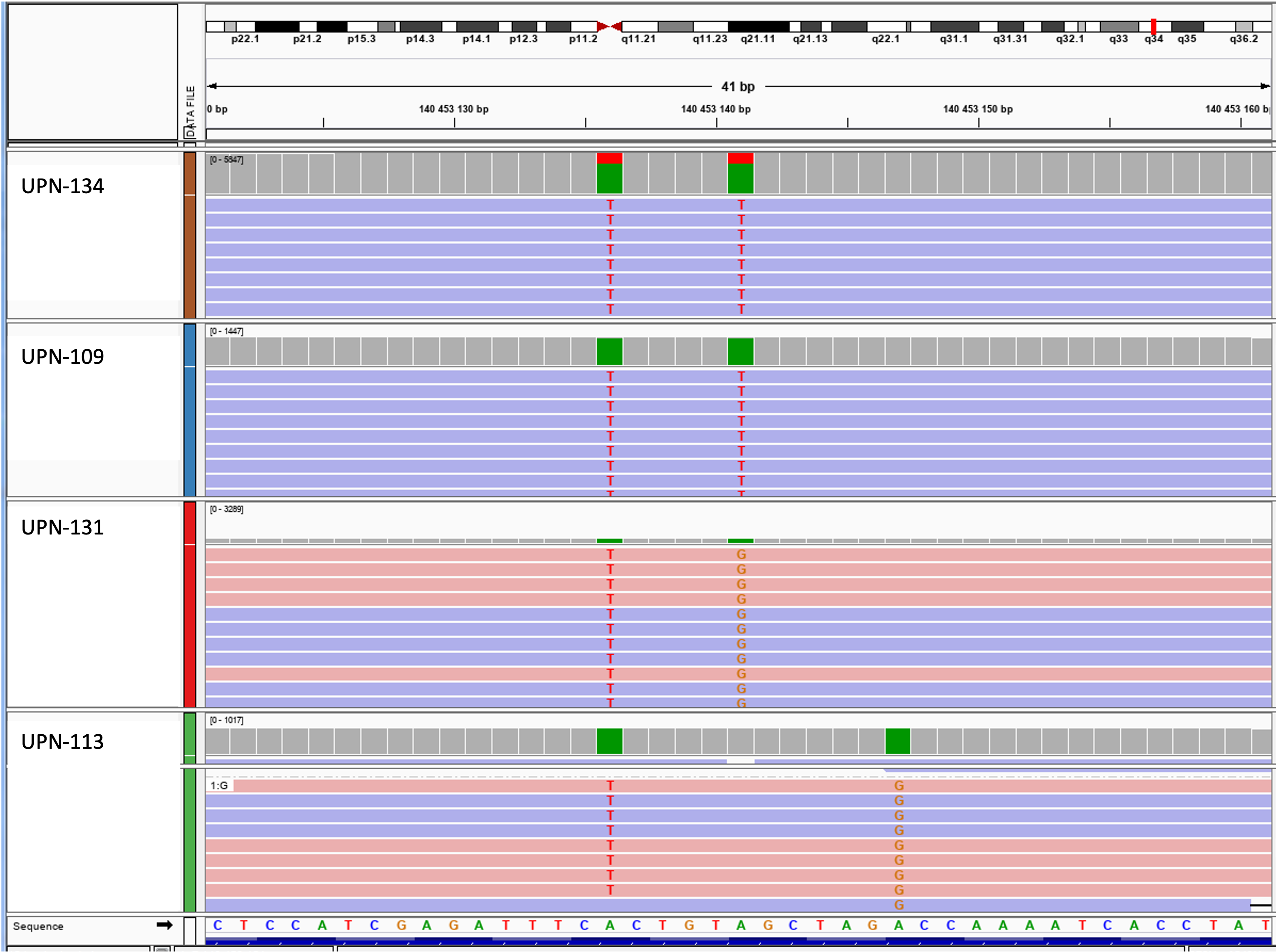

Supplement: Supplementary file 4 — Figure S4 [file JCMM-27-2626-s004.png]
