# Supplementary material for: Hairy cell leukaemia with unusual BRAF mutations
Source: J Cell Mol Med. 2023 Aug 2;27(17):2626–30. doi: 10.1111/jcmm.17890 (PMC10468650; doi:10.1111/jcmm.17890)
Supplement: Supplementary file 5 — Table S1 [file JCMM-27-2626-s003.docx]

Supplementary table

List of the 21-panel genes

| Gene | Chromosomic location | Exons | RefSeq (NCBI) |
| --- | --- | --- | --- |
| *BRAF* | 7q34 | 15 et 11 | NM_004333 |
| *MAP2K1* | 15q22.31 | 2-3 | NM_002755.2 |
| *MAPK15* | 8q24.3 | 1-14 | NM_139021 |
| *DUSP2* | 2q11.2 | 1-4 | NM_004418 |
| *ARID1A* | 1p36.11 | 1-20 | NM_006015 |
| *ARID1B* | 6q25.3 | 1-20 | NM_020732 |
| *CREBBP* | 16p13.3 | 1-31 | NM_004380 |
| *EZH2* | 7q36.1 | 2-20 | NM_004456 |
| *KDM6A* | Xp11.3 | 1-29 | NM_021140 |
| *CDKN1B* | 12p13.1 | 1-2 | NM_004064 |
| *TP53* | 17p13.1 | 2-11 | NM_000546 |
| *ANXA1* | 9q21.13 | 2-13 | NM_000700 |
| *BCOR* | Xp11.4 | 2-15 | NM_017745 |
| *U2AF1* | 21q22.3 | 2 et 6 | NM_001025203/NM_006758 |
| *MYD88* | 3p22.2 | 3-5 | NM_002468 |
| *CXCR4* | 2q22.1 | 1 | NM_001008540 |
| *KLF2* | 19p13.11 | 1-3 | NM_016270 |
| *ABCA8* | 17q24.2 | 2-38 | NM_007168 |
| *XPO1* | 2p15 | 15-18 | NM_003400 |
| *NOTCH1* | 9q34.3 | 26-27-34 | NM_017617 |
| *NOTCH2* | 1p12 | 26-27-34 | NM_024408 |
